# Treatment of major vein injury with the hemostatic fleece TachoSil by interposing a peritoneal patch to avoid vein thrombosis: A feasibility study in pigs

**DOI:** 10.4103/0974-2700.76822

**Published:** 2011

**Authors:** Einar B Dregelid, Gustav Pedersen

**Affiliations:** 1 Department of Vascular Surgery, Haukeland University Hospital, Norway; 2 Institute for Surgical Sciences, University of Bergen, Jonas Lies vei 65, 5021 Bergen, Norway

**Keywords:** Hemostatic agent, Tachosil, vascular trauma, vein injury, vein repair

## Abstract

**Background:**

Vein lacerations in awkward locations are difficult to repair and carry high mortality. The hemostatic fleece, TachoSil, is effective in preventing intraoperative bleeding in different settings, but has not been recommended for use in large vein injury. TachoSil with a peritoneal patch interposed to avoid vein thrombosis has been reported as a method to obtain hemostasis in vein laceration, but further studies of this method are needed.

**Materials and Methods:**

A 1.5 × 1 cm defect was created in the vena cava in five pigs. A 26 × 32 mm peritoneal patch was applied on the coagulant side of a 48 × 48 mm TachoSil sheet, and used to cover the defect. Light compression with a wet sponge was applied for 3 min. No vascular suturing was performed.

**Results:**

Successful hemostasis was obtained in four out of the five pigs although the minimum TachoSil gluing zone surrounding the peritoneal patch was only 0–2 mm. The fifth pig died of hemorrhage 30 min after surgery due to a 4-mm stretch with no TachoSil gluing zone outside the peritoneal patch. At six days postoperatively the peritoneal patch was well integrated into the vein wall. After 28 days, the peritoneal patch was almost indiscernible from surrounding vein endothelium.

**Conclusions:**

Vein wall defects can be repaired using TachoSil with a peritoneal patch interposed to prevent contact between the thrombogenic TachoSil sheet and the vein lumen. An adequate TachoSil gluing zone all around the patch is essential.

## INTRODUCTION

Vein lacerations in awkward locations are difficult to repair with vascular sutures, and carry high mortality.[1–3] Hemostatic fleeces like TachoSil (Nycomed Austria GmbH, Linz, Austria), can provide effective intraoperative hemostasis in different settings and adhere firmly to moist tissues, even in hypocoagulable patients, but have not been recommended for use in large vein injury.[4–8] TachoSil with a peritoneal patch interposed to prevent vein thrombosis has been reported as a method to obtain hemostasis in vein laceration, but documentation is scarce.[9,10] In this investigation, we evaluated the feasibility of using a peritoneal patch and TachoSil to repair partial wall defects in the vena cava of pigs.

## MATERIALS AND METHODS

### Animal handling and anesthesia

Five domestic pigs (*Sus scrofa domestica, Norwegian Landrace–Yorkshire hybrid*), three months old, with initial weight 37 kg (range: 34–40) of either sex were used for the experiments. The animals were on a standard pig feed (Format Norm 106n, Felleskjøpet AGRI, Oslo, Norway), were kept under controlled environmental conditions, and were acclimatized at the facility for a minimum of five days before the experiments. Food was withdrawn 8 to 12 h before anesthesia. Water was available at all times. The study protocol was approved by the local ethical committee for animal care and use, and all experiments were conducted in compliance with institutional guidelines.

The animals were premedicated with a subcutaneous injection of ketamine hydrochloride, 12 mg/kg (Ketalar, Pfizer Inc., NY), Medetomidine hydrochloride, 0.12 mg/kg (range 0.1–0.19) (Domitor, Orion Corporation, Turku, Finland), and atropine, 0.03 mg/kg (Nycomed Pharma AS, Oslo, Norway) subcutaneously. Prokainpenicillin, 22 mg/kg and dihydrostreptomycin, 27.5 mg/kg (Streptocillin vet., Boehringer Ingelheim Danmark A/S, Copenhagen, Denmark) was injected intramuscularly. Induction with 1–1.2% isoflurane (Isoba vet., Schering-Plough Animal Health Corporation Union, NJ) in a mixture of 50% oxygen and 50% air was performed before intubation of the trachea, and maintenance of anesthesia was achieved by ventilation with 1–1.2% isoflurane in a mixture of 44% oxygen and 56% N_2_ O. Morphine 2 mg/kg (Nycomed Pharma AS, Oslo, Norway) was given intravenously before starting the operation. Temperature, pulse, oxygenation, and heart rate were monitored using electrocardiography and pulse oximetry on an ear or lip and on the tail (Rad-5v, Masimo Corporation, CA). During the operation, Ringer’s acetate solution was given at the rate of 11 ml/kg/h (range: 5.4–11.4) and the animals were lying on a warming mattress.

Immediately postoperatively, Doxapram hydrochloride, 0.6 mg/kg (range: 0.5–1.16) (Dopram, Meda A/S, Oslo, Norway) and Atipamezolhydrochlorid, 0.025 mg/kg (range: 0.013–0.14) (Antisedan vet., Orion Corporation, Turku, Finland) were given intravenously to enhance recovery of spontaneous respiration. Morphine in doses of 2 mg/kg was given intramuscularly when the animals recovered spontaneous respiration after the operation and additional doses of 1 mg/kg were given during the first 24 h when the animals seemed to be affected by pain.

### Surgical procedures

**Figure 1:**
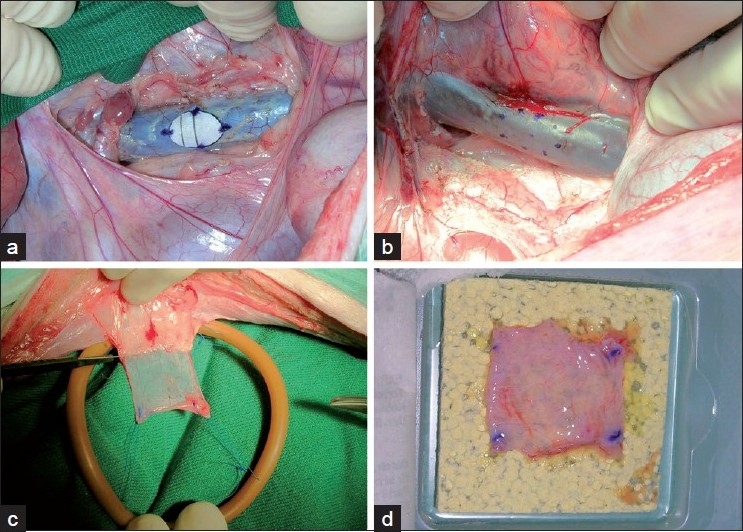
Cava portion to be excised (panels a and b), peritoneal patch harvesting (panel c), and centering of patch on TachoSil sheet (panel d)

A 32 cm (range: 30–34) midline laparotomy was performed. The inferior vena cava was exposed distal to the right renal vein. An elliptical portion, 1.5 cm long and 1 cm wide, corresponding to a sterilized elliptical paper mold was excised from the anterior aspect of the vena cava using a knife and scissors [Figure 1a and b]. A patch of parietal peritoneum, measuring 26 × 32 mm, corresponding to a sterilized paper mold, was excised from the margin of the laparotomy and put in 0.9% saline solution. A surgical marker pen (Accu-line Surgical Marking Pen, Accu-line Products, Inc. MA) was used to mark the size of the peritoneal patch and the size of the portion of caval wall that was to be excised. Subsequently, the peritoneal patch was applied on the coagulant side of a 48 × 48-mm TachoSil sheet, which was then used to cover the caval lesion with the peritoneal side of the patch facing the lumen. A wet sponge was used for light compression for three minutes. Finger compression was used to occlude the vena cava until the sponge had been applied.

The abdominal wound was sutured using running sutures of Vicryl 1-0 for the fascia and Nylon 3-0 skin suture intradermally (both: Ethicon, Johnson and Johnson Intl, Somerville, NJ). The animals were allowed to walk and feed freely. After six days and four weeks a relaparotomy was performed. The injured portion of the vena cava, including 1 cm proximally and distally, was excised and put in formaldehyde solution.

Animals surviving until elective excision of the reconstructed portion of the vena cava were euthanized by i.v. injection of a 20 ml bolus of saturated potassium chloride. All animals underwent a partial autopsy. The vena cava, heart chambers, pulmonary and coronary arteries, and aorta were opened and inspected visually.

### Processing and examination of specimens

The injured portion of the vena cava, including one cm proximally and distally, was excised, rinsed gently with heparinized saline and put in formaldehyde solution. It was then cut open dorsally, unfolded and fixed to a plate of expanded polystyrene using needles, and subjected to further fixation. The luminal surface of the vena cava with the luminal surface of the patch was inspected and photographed. After fixation for 8-10 days in formaldehyde solution, the specimens were transferred to ethanol. The TachoSil sheet was carefully dissected off from the vein and the minimum width of the TachoSil sheet that was not covered by the peritoneal patch, and hence constituted the minimum gluing zone, was measured.

## RESULTS

### Handling of the peritoneal patch

In the first two animals we experienced that it was somewhat difficult to pick the rolled-up patch of peritoneum from the saline solution and immediately hit the center of the TachoSil sheet with the center of the peritoneal patch. The coagulant agent on the TachoSil is immediately activated on contact with the peritoneal patch, which therefore has to be placed in the right position in the first place. From the third pig onwards we therefore harvested the peritoneal patch using stay sutures fixed to a circular rubber frame to hold the peritoneal patch unfolded. The frame was made using a 24.7 cm segment from an 18 F Foley catheter (Bard Limited, Crawley, UK). The two ends of the segment were connected using two pieces of a match as a splint in the lumen of the catheter [Figure 1c]. The frame with the patch was then put in the saline solution. Subsequently, the frame with the patch suspended in its center was placed on the TachoSil sheet, thus making it easy to align the center of the patch with that of the TachoSil. The stay sutures were then cut and the frame removed [Figure 1d].

### Change in fluid volume and drug administration

Since a rash in pig number 1 appeared after injection of Antisedan, the dose of this drug was reduced for the following animals. Extravasation of fluid in the skin rash may have caused hypovolemia. Periods of tachycardia immediately postoperatively are compatible with this hypothesis. Since pig number 2, which was operated on the same day as pig number 1 and given the same doses of analgesics, was affected by wound pain the morning after the operation, it is likely that pig number 1 too had suffered from pain. It probably died of subendocardial infarction due to a combination of hypovolemia and stress from wound pain (see account of pig number 1 in the next section). From pig number 3 onwards we therefore administered more fluid during the operation, and another dose of morphine was administered postoperatively to pig numbers 4 and 5.

### The fate of each experimental animal and vein repair

**Figure 2:**
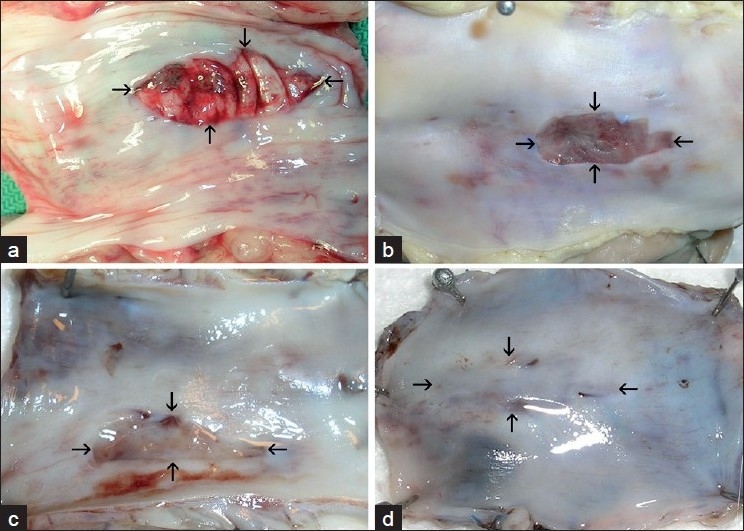
Peritoneal patches in the wall of the vena cava 6, 5–21 h (panel a, pig number 1), 6 days (panel b, pig number 4 and panel c, pig number 5), and 28 days (panel d, pig number 2) after vein repair

**Figure 3:**
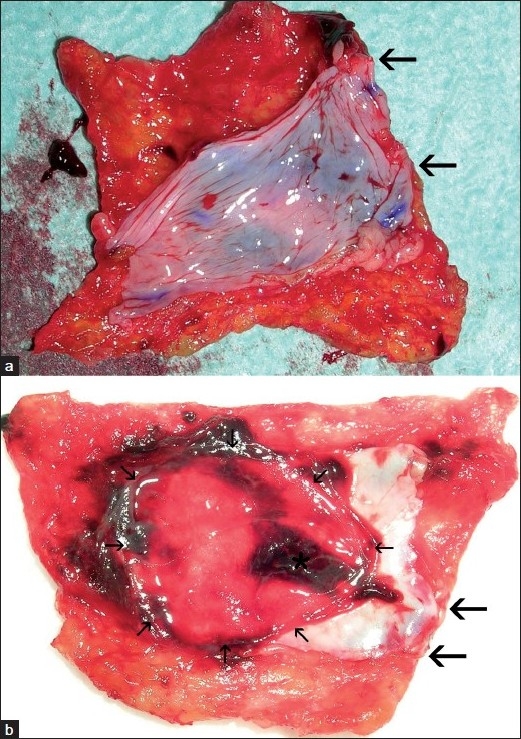
First (panel a) and second (panel b) TachoSil-peritoneal patch harvested from pig number 3 that bled to death. Large arrows demarcate stretches without gluing zones. Small arrows demarcate portion of the peritoneum corresponding to the caval defect. Asterisk marks thrombus

Vein repair was successful in 4 of 5 animals despite the fact that the minimum width of the TachoSil sheet outside the peritoneal patch was 0 mm in two animals, including one that bled to death half an hour postoperatively, 1 mm in two animals and 2 mm in one [Table 1]. Figure 2 displays successful vein repairs and Figure 3 unsuccessful repairs as seen from the luminal side of the vena cava. The photographs of the specimens shown in Figures 2a and 3a and b are taken before, and those of the specimens shown in Figure 2b–d are taken after fixation. The arrows in Figure 2 show the border between peritoneal patch and vein. No intracardiac or intravascular thrombi were found at autopsies with the exception of a few thrombus strands adhering to the trabeculae of the right auricular appendage of pig number 3.

**Table 1:** The fate of each experimental animal and vein repair

Pig number*	Repair observation time†	Repair outcome	Minimum TachoSil gluing zone (mm)	Blood squirt after compression	Mode of death
1	6.5–21 h	Intact	0	No	Myocardial Infarction
2	28 days	Endothelialized	1	Yes	Euthanized
3	30 min	Failure	0	Yes	Hemorrhage
4	6 days	Intact	2	Yes	Wound Rupture
5	6 days	Intact	1	No	Euthanized

* DENOTES THE SEQUENCE OF THE EXPERIMENTS

† TIME TO DEATH AFTER VEIN REPAIR

Pig number 1 had a slow recovery after anesthesia. Its skin attained a pink mottled rash after the injection of Antisedan. It had periods of tachycardia with heart rate up to 182. When left unattended in the afternoon 6.5 h after the operation, it had drunk and eaten, and had been standing, but its balance was unsteady. The next morning, 21 h after the operation, it was found dead. At a partial autopsy, no blood was found in the abdomen; the TachoSil and peritoneal patch adhered firmly to the opening that had been made in the vena cava [Figure 2a]. There were signs of subendocardial hemorrhages, indicating subendocardial infarction, in the outflow portion of the left ventricle.

In pig number 2, there was ballooning of the TachoSil sheet and initially a squirt of blood from one side of the TachoSil sheet when the saline sponge used for compression was removed after three minutes of light compression. After another period of compression for about two minutes, no bleeding was seen, and the abdomen was closed. The animal was healthy until it was euthanized at 28 days. The peritoneal patch had become integrated into the vein wall and was virtually indiscernible from surrounding vein endothelium [Figure 2d].

In pig number 3, following removal of the wet sponge after three minutes of light compression, there was almost immediately brisk bleeding from the caudal side of the TachoSil sheet, which was then removed together with the peritoneal patch [Figure 3a]. The peritoneal patch extended to the margin of the TachoSil sheet along the caudal side for a 12-mm distance (between the arrows in Figure 3a). A new TachoSil sheet with a new peritoneal patch was applied, this time with initial success, although there was a small squirt of blood that subsided after some more compression as in pig number 2. The pig bled to death, however, about 30 min after the surgical procedure, just after waking up from the anesthesia. Autopsy showed a copious amount of blood in the peritoneal cavity. The bleeding again had occurred from the caudal margin of the cava repair where the peritoneal patch extended to the margin of the TachoSil sheet for a 4-mm stretch (between the thick arrows in Figure 3b). Hence, along the 4-12 mm stretches between the thick arrows in Figure 3a and b there were no gluing zones outside the peritoneal patches, explaining why hemostasis was not obtained with the first repair, and lasted for only 30 min after the second repair. It appeared that the TachoSil sheet had been crimped during application. In Figure 3b the area indicated with small arrows is the portion of the peritoneum corresponding to the oval defect in the vena cava. The asterisk marks mural thrombus on the portion of the peritoneal patch adjacent to the margin where the blood had escaped from the vena cava due to lack of a gluing zone.

In pig number 4 there was also a small squirt of blood from the caudal side of the TachoSil sheet when the wet sponge that had been used for compression was removed. We therefore elected to apply one half of a small TachoSil sheet in addition, to seal this side. This pig was found dead with a wound rupture in the morning, the sixth day after the operation. The day before, the pig had been healthy with no sign of hernia. At autopsy, the cava repair was intact [Figure 2b].

Pig number 5 was healthy, apart from a serous fluid collection in the wound, until it was euthanized at 6 days. The cava repair was intact and totally covered with firmly adherent surrounding anatomical structures [Figure 2c].

## DISCUSSION

The most important finding in this study is that when a 1.5 × 1 cm large defect in the vena cava was covered with a 26 × 32 mm peritoneal patch and a 48 × 48 mm TachoSil sheet, only supplied with an extra half sheet in one animal, hemostasis was obtained in four out of five pigs. No vascular suturing was performed. These results were obtained despite the fact that focally there was no TachoSil gluing zone outside the peritoneal patch in two animals, one of which died of hemorrhage 30 min after vein repair. In the remaining three, the minimum gluing zone was 1–2 mm. A previous study has shown that a peritoneal tube may successfully replace a 2.5 cm segment of the vena cava, but longer tubes will undergo thrombotic occlusion.[10] Vein wall defects longer than 2.5 cm may therefore not be amenable to this repair technique.

We think the explanation of the absent or narrow gluing zones is that the distance between the fingers used for compression of the vena cava above and below the lesion was too short. It appears that if a TachoSil sheet is crimped during application it will remain so due to the fibrin glue and cannot be stretched again. There has to be enough space for the entire TachoSil sheet between fingers or vascular clamps that are used for vein occlusion during application. If it is not possible to provide enough space, additional TachoSil should be applied after the initial compression period to secure a sufficient gluing zone outside the peritoneal patch, according to the producer’s recommendation. We deliberately selected the smallest TachoSil sheet, measuring 48 × 48 mm, to test the limits of the method. With a peritoneal patch measuring 26 × 32 mm, this resulted in a gluing zone of maximally 8 mm cranially and caudally only if the peritoneal patch could be put in the centre of the TachoSil sheet and no crimping of the TachoSil sheet occurred.

A squirt of blood from the TachoSil margin on removal of the sponge heralded the fatal postoperative bleeding in pig number three but was also observed in pig numbers 2 and 4 corresponding to the margins with narrow or absent gluing zones. A squirt of blood on termination of light compression therefore may indicate an insufficient gluing zone and a risk of subsequent bleeding. Since there were absent or narrow gluing zones also in pig numbers 1 and 5, the absence of a squirt of blood after compression does not, however, guarantee a sufficiently safe gluing zone.

The mural thrombus that was seen on the peritoneal patch adjacent to the margin where the bleeding had occurred in pig number 3 [Figure 2b], probably was due to tissue factor activation of the coagulation system as a consequence of the copious bleeding.[11] On the peritoneal patch of pig number 1 that died 6.5–21 h after vein repair there was only patchy mural thrombus. At 6 and 28 days, the patches were free from thrombus [Figure 2a–d]. These findings are consistent with previous findings that fibrinolytic activity of autologous peritoneum first are impaired, and then increase after a trauma, reaching higher values than those of normal endothelium four days after transplantation to a vein.[12] Despite a probable fibrinolysis activity on the peritoneal patch, vein repair was firm, and after 6 days the patch was well integrated into the vein wall.

## CONCLUSIONS

This study and a previous case report, show that it is feasible to repair large vein defects using TachoSil with a peritoneal patch interposed to prevent contact between the coagulant-containing side of the TachoSil sheet and the vein lumen.[9] Vein wall defects longer than 2.5 cm may not be amenable to this repair technique.[10] Although a minimum gluing zone of 0 to 2 mm provided successful hemostasis in four of our five experimental animals, a broader zone all around the patch is necessary for safe hemostasis. An insufficient gluing zone may be present wherever a squirt of blood appears on removal of the sponge used for compression, but the absence of a squirt of blood after the compression period does not exclude the presence of a narrow gluing zone.

## References

[1] Asensio JA, Petrone P, Garcia-Nuñez L, Healy M, Martin M, Kuncir E (2007). Superior mesenteric venous injuries: To ligate or to repair remains the question. J Trauma.

[2] Coimbra R, Filho AR, Nesser RA, Rasslan S (2004). Outcome from traumatic injury of the portal and superior mesenteric veins. Vasc Endovascular Surg.

[3] Oderich GS, Panneton JM, Hofer J, Bower TC, Cherry KJ, Sullivan T (2004). Iatrogenic operative injuries of abdominal and pelvic veins: A potentially lethal complication. J Vasc Surg.

[4] Siemer S, Lahme S, Altziebler S, Machtens S, Strohmaier W, Wechsel HW (2007). Efficacy and safety of TachoSil as haemostatic treatment versus standard suturing in kidney tumour resection: A randomised prospective study. Eur Urol.

[5] Carbon RT, Baar S, Waldschmidt J, Huemmer HP, Simon SI (2002). Innovative minimally invasive pediatric surgery is of therapeutic value for splenic injury. J Pediatr Surg.

[6] Kudo M, Misumi T, Koizumi K, Shin H (2005). A surgical case of ventricular septal perforation after repairing left ventricular free wall rupture. Ann Thorac Cardiovasc Surg.

[7] Joseph T, Adeosun A, Paes T, Bahal V (2004). Randomised controlled trial to evaluate the efficacy of TachoComb H patches in controlling PTFE suture-hole bleeding. Eur J Vasc Endovasc Surg.

[8] Carbon RT, Schmidt A, Baar S, Kriegelstein S, Faist E (2004). Tissue Management with Fleece-Bound Sealing. 6th World Congress on Trauma, Shock, Inflammation and Sepsis - Pathophysiology, Immune Consequences and Therapy.

[9] Dregelid E, Ramnefjell MP, Erichsen C, Christensen BJ, Rawal R (2008). Effective hemostasis in severe mesenteric vein laceration with Tachosil (R), using a low- or non-thrombogenic patch to prevent Tachosil (R)-induced thrombosis. Eur J Trauma Emerg Surg.

[10] Ribbe EB, Alm P, Hallberg E, Norgren LE (1988). Evaluation of peritoneal tube grafts in the inferior vena cava of the pig. Br J Surg.

[11] Ohan J, Leseche G, Gilbert MA, Trugnan G, Drouet L (2001). Human mesothelial cells express tissue factor when switched to proliferating state: Pharmacological modulation *in vitro*. Blood Coagul Fibrinolysis.

[12] Louagie Y, Legrand-Monsieur A, Remacle C, Maldague P, Lambotte L, Ponlot R (1986). Morphology and fibrinolytic activity of canine autogenous mesothelium used as venous substitute. Res Exp Med (Berl).

